# Effect of food on the pharmacokinetics of omeprazole, pantoprazole and rabeprazole

**DOI:** 10.1186/s40360-020-00433-2

**Published:** 2020-07-25

**Authors:** Dolores Ochoa, Manuel Román, Teresa Cabaleiro, Miriam Saiz-Rodríguez, Gina Mejía, Francisco Abad-Santos

**Affiliations:** 1 Clinical Pharmacology Department, Hospital Universitario de La Princesa, Instituto Teófilo Hernando, Universidad Autónoma de Madrid (UAM), Instituto de Investigación Sanitaria La Princesa (IP), C/Diego de León 62, 28006 Madrid, Spain; 2 UICEC Hospital Universitario de La Princesa, Plataforma SCReN (Spanish Clinical Reseach Network), Instituto de Investigación Sanitaria La Princesa (IP), Madrid, Spain; 3 Research Unit, Fundación Burgos por la Investigación de la Salud, Hospital Universitario de Burgos, Burgos, Spain; 4 Pharmacology Department, Facultad de Medicina, Universidad Autónoma de Madrid, Madrid, Spain

**Keywords:** Proton pump inhibitors, Food, Pharmacokinetics, Omeprazole, Rabeprazole, Pantoprazole

## Abstract

**Background:**

The pharmacokinetics of proton pump inhibitors (PPIs) may be affected by food intake. We aimed to evaluate the effect of food on the pharmacokinetics of omeprazole, rabeprazole, and pantoprazole.

**Setting:**

The study population comprised 186 healthy volunteers participating in 6 bioequivalence clinical trials.

**Method:**

Subjects were evaluated to determine the effect of a high-fat breakfast on the pharmacokinetics of omeprazole (*n* = 36), rabeprazole (*n* = 69), and pantoprazole (*n* = 81).

**Main outcome measure:**

Drug plasma concentrations were measured using high-performance liquid chromatography coupled to mass spectrometry.

**Results:**

Food affected the pharmacokinetics of omeprazole (increased T_max_ and decreased AUC and C_max_), pantoprazole (increased T_max_ and decreased AUC), and rabeprazole (increased T_max_, C_max_ and half-life). Food increased variability in T_max_ for all 3 drugs, delaying absorption around 3 to 4 h and until 20 h in some subjects.

**Conclusion:**

As food delays the absorption of PPIs and increases their variability, it would be better to administer these drugs under fasting conditions.

**Trial registration:**

European Union Drug Regulating Authorities Clinical Trials Database: EudraCT: 2004–003863-59 (registration date 05/MAR/2004), EudraCT 2006–001162-17 (registration date 17-MAR-2006), EudraCT: 2007–002489-37 (registration date 12-JUN-2007), EudraCT: 2007–002490-31 (registration date 12-JUN-2007), EudraCT: 2010–024029-19 (registration date 23-NOV-2010).

## Bulleted statements on the impact of the research findings on patients, pharmacy or clinical practice

1. To be more effective, it is recommended to administer PPIs better under fasting conditions.
2. More effective administration could improve patient outcomes.

## Background

Proton pump inhibitors (PPIs) are used for the treatment of acid-related diseases such as gastric and duodenal ulcers, gastroesophageal reflux disease, non-erosive reflux disease, and Zollinger-Ellison syndrome. They are also used in combination with antibiotics for the eradication of *Helicobacter pylori* [1].

PPIs inhibit gastric H^+^/K^+^-ATPase pump activity, resulting in potent acid inhibition. However, the ability of PPIs to suppress gastric acid varies widely between individuals. Several factors contribute to this phenomenon, including the considerable variation in the oral bioavailability of PPIs, the need for the ATPase pumps to be activated by food, the influence of *Helicobacter pylori*–associated gastritis, and genetic variation in enzyme activity [2]. Indeed, drug formulation, food intake, and single-nucleotide polymorphisms in cytochrome P450 (CYP) *2C19* enzyme also influence PPIs pharmacokinetic parameters [3–5]. Thus, the time to maximum plasma concentration (T_max_) varies from 1 h to 5 h depending on the drug formulation and/or food effect [3, 4].

Tytgat et al. reported that the bioavailability of PPIs was markedly affected by food intake [6]; consequently, PPIs should be taken under fasting conditions. However, PPIs prescribing information is controversial, since some authors state that in case of omeprazole and rabeprazole the concomitant ingestion of food does not affect their bioavailability [7, 8], whereas others state that omeprazole should be taken at least 1 h before a meal [9]. The recommendations for pantoprazole depend on the formulation: tablets could be taken regardless of meal timing, but oral suspension should be taken 30 min before a meal [10]. To date, there was some evidence that taking PPIs with food affected their absorption. However, until now it has not been rigorously evaluated. This study provides the necessary evidence.

## Aim of the study

This study aimed to evaluate the influence of food on the pharmacokinetic parameters of three PPIs (omeprazole, rabeprazole and pantoprazole) and to determine whether food is more relevant for any of them.

## Methods

### Study design

The study population comprised 186 healthy Caucasian adult volunteers from 6 single-dose bioequivalence clinical trials with pantoprazole (40 mg, enteric-coated tablets), rabeprazole (20 mg, enteric-coated tablets), and omeprazole (40 mg, oral capsules). The bioequivalence clinical trials were phase I, randomized, open-label, crossover, single-centre, with two periods separated by a 7-day washout stage. Subjects were randomised to one of the two treatment sequences, RT or TR where R was the reference formulation and T was the test formulation. Each of the clinical trial was crossover for evaluation of a test formulation compared to a reference formulation either under fed or fasting conditions. Different subjects participated in each trial, but in case of omeprazole, that 35 of 36 volunteers were the same for the trials under fed and fasting conditions. The treatments were allocated in a balanced manner on every inclusion day (blocks of 4 subjects). Our study adheres to CONSORT guidelines.

Considering the expected intrasubject variability for each study, using a multiplicative model, sample size was calculated to reject a difference between both formulations of 20%, with a power of 80% and an alpha error of 0.05, according to the bioequivalence approaches habitually accepted by the Health Authorities (acceptance limits of 0.8–1.25).

From those 186 healthy volunteers, 81 subjects received pantoprazole (36 under fasting conditions, EudraCT: 2004–003863-59; and 45 with food, EudraCT 2006–001162-17), 69 received rabeprazole (35 under fasting conditions, EudraCT: 2007–002489-37; and 34 with food, EudraCT: 2007–002490-31) and 36 received omeprazole (35 under fasting conditions and 36 with food; EudraCT: 2010–024029-19). In case of omeprazole, 35 volunteers were the same for both trials. In the trials with food, volunteers fasted for 10 h, the breakfast was taken in 20 min (between 30 and 10 min previous to dosing) and the drug was administered 10 min after the end of breakfast. The meal had a high fat content (50–60% of total caloric content of the meal) and high calorie count (approximately 800 to 1000 kcal) according to EMA [11] and FDA [12] guidelines.

The inclusion criteria were as follows: non-smoking male and female volunteers, age 18 to 55 years, body mass index (BMI) within the 18.5–30.0 range, free from any organic or psychiatric conditions, no taking any drug and with normal vital signs, electrocardiogram (ECG), medical records and physical examination. It was not allowed to take other drugs during the study.

### Sample processing, quantification, and pharmacokinetic analysis

Sampling was extended for 12 h in the clinical trials under fasting conditions and for 24 h under fed conditions, as follows: (i) Fasting omeprazole – 21 samples: predose, 0.33, 0.67, 1, 1.33, 1.67, 2, 2.33, 2.67, 3, 3.33, 3.67, 4, 4.5, 5, 5.5, 6, 7, 8, 10 and 12 h; (ii) fed omeprazole - 26 samples: predose, 0.5, 1, 1.5, 2, 2.5, 3, 3.5, 4, 4.5, 5, 5.5, 6, 6.5, 7, 7.5, 8, 8.5, 9, 10, 1 h, 12, 14, 17, 20 and 24 h; (iii) fasting pantoprazole – 14 samples: predose, 0.5, 1, 1.5, 2, 2.5, 3, 3.5, 4, 5, 6, 8, 10 and 12 h; (iv) fed pantoprazole – 37 samples: predose, 1, 1.5, 2, 2.5, 3, 3.5, 4, 4.5, 5, 5.5, 6, 6.5, 7, 7.5, 8, 8.5, 9, 9.5, 10, 10.5, 11, 11.5, 12, 12.5, 13, 13.5, 14, 15, 16, 17, 18, 19, 20, 21, 22 and 24 h; (v) fasting rabeprazole – 15 samples: predose, 1, 1.5, 2, 2.5, 3, 3.5, 4, 4.5, 5, 6, 7, 8, 10 and 12 h; (vi) fed rabeprazole – 37 samples: predose, 1, 1.5, 2, 2.5, 3, 3.5, 4, 4.5, 5, 5.5, 6, 6.5, 7, 7.5, 8, 8.5, 9, 9.5, 10, 10.5, 11, 11.5, 12, 12.5, 13, 13.5, 14, 15, 16, 17, 18, 19, 20, 21, 22 and 24 h.

Sample processing, genotyping for CYP2C19 and drug quantification were performed according to Román et al. [5]. As the formulations were bioequivalent, we used the mean concentrations obtained after receiving both test and reference formulations to calculate the pharmacokinetic parameters of each subject. Pharmacokinetic parameters were estimated from the plasma concentration–time data by non-compartmental analysis (WinNonlin Professional, version 2.0., Pharsight Corporation, USA) as reported by Román et al. [5].

### Data analysis

WinNonLin Professional software version 2.0 was used for the statistical analysis. Pharmacokinetic parameters were log-transformed, and AUC and C_max_ were adjusted for dose and weight. The values of pharmacokinetic parameters were expressed as mean ± standard deviation. An analysis of the variance (ANOVA) test was applied to calculate the statistical significance of the differences in pharmacokinetic parameters considering the factors sex and food condition; in the model for omeprazole the factor subject was also included. The 90% confidence interval of the ratio of geometric means between fed and fast conditions were calculated. To avoid the influence of *CYP2C19* polymorphisms, this analysis was repeated in CYP2C19*1/*1 subjects. *p* ≤ 0.05 was considered significant.

## Results

### Study population

We analyzed 186 volunteers (95 men and 91 women). Average age was higher in the omeprazole study (26.73 ± 5.74 years) than in the pantoprazole study (23.81 ± 3.18 years, *p* ≤ 0.001) and the rabeprazole study (24.62 ± 3.78 years, *p* ≤ 0.05). Weight was similar in the three drugs clinical trials (66.25 ± 11.27 kg for pantoprazole, 68.21 ± 13.07 kg for rabeprazole, and 67.69 ± 12.78 kg for omeprazole).

Thirty five subjects from omeprazole study accepted CYP2C19 genotyping, being 16 *1/*1, 7 *1/*2, 1 *2/*2, 10 *1/*17 and 1 *2/*17. In the case of pantopazole, 33 subjects were genotyped in the fasting trial (14 *1/*1, 6 *1/*2, 11 *1/*17 and 2 *17*17) and 36 in the fed trial (14 *1/*1, 6 *1/*2, 14 *1/*17, 1 *17/*17 and 1 *2/*17). In the rabeprazole studies, 30 subjects were genotyped in the fasting trial (14 *1/*1, 5 *1/*2, 9 *1/*17 and 2 *2/*17) and 22 in the fed trial (9 *1/*1, 4 *1/*2, 8 *1/*17 and 1 *2/*17).

### Role of food in the pharmacokinetics of PPIs

**Fig. 1:**
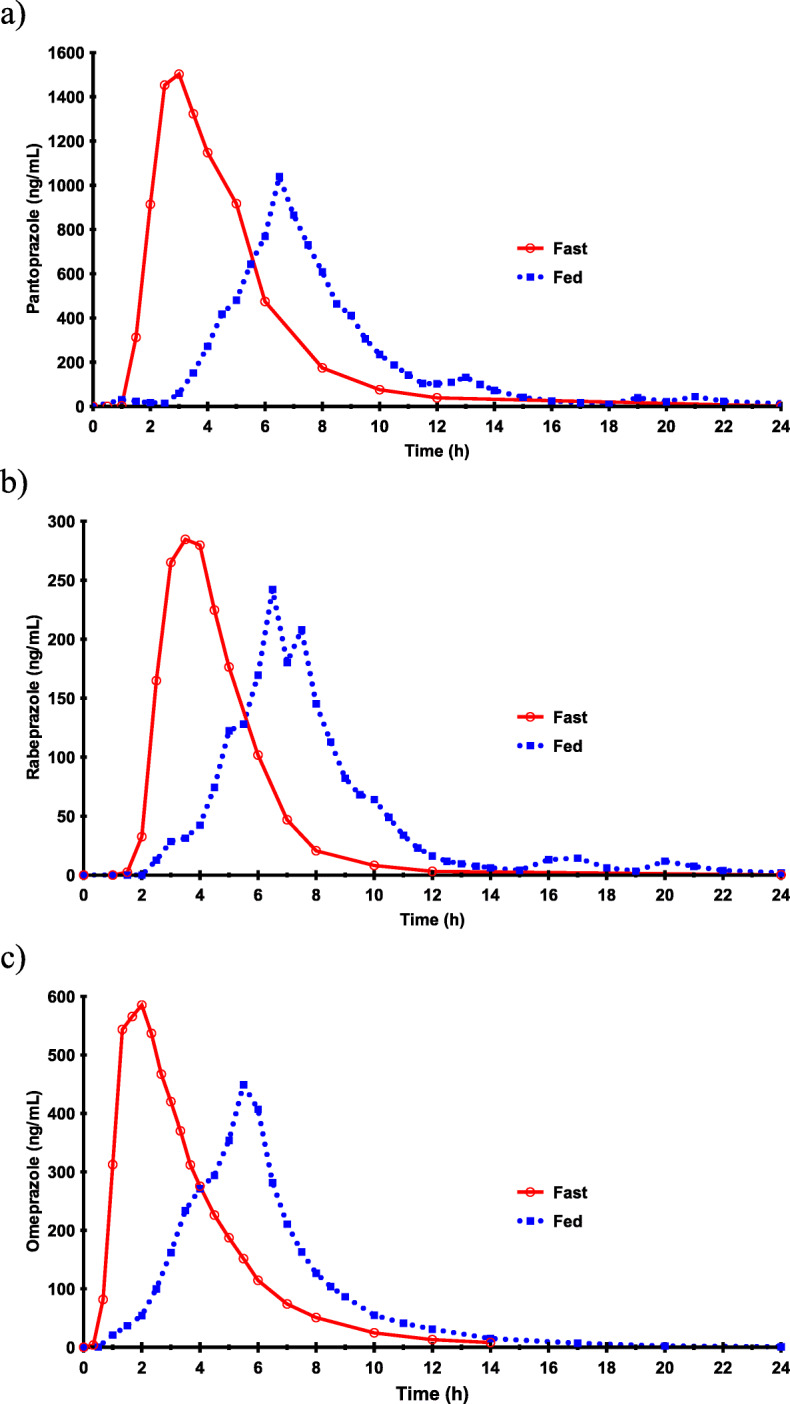
Mean plasma concentration-time profiles of (**a**) pantoprazole, (**b**) rabeprazole and (c) omeprazole, when administered under fed and fasting conditions

The effect of food on the pharmacokinetics of omeprazole, pantoprazole, and rabeprazole is shown in Table 1. Mean plasma concentration-time profiles are depicted in Fig. 1.

**Table 1:** Pharmacokinetic parameters for pantoprazole, rabeprazole, and omeprazole under fasting and fed conditions. Among 71 volunteers receiving omeprazole, 35 were the same subjects. Data are expressed as mean ± standard deviation. ^*^p ≤ 0.05 vs. men

	n	AUC_0-∞_ (ng·h/ml)	C_max_ (ng/ml)	T_max_ (h)	t_**1/2**_ (h)	Vd/F (l/kg)	Cl/F (L/h·kg)	Ke
**Pantoprazole (n = 81)**								
**Fast**	36	6951.4 ± 3293.2	2779.1 ± 861.6	3.0 ± 0.8	1.3 ± 0.6	0.17 ± 0.03	0.11 ± 0.04	0.61 ± 0.21
Men	18	6584.5 ± 3832.8	2316.4 ± 580.9	3.1 ± 0.8	1.5 ± 0.7	0.18 ± 0.03	0.11 ± 0.03	0.56 ± 0.23
Women	18	7318.4 ± 2711.6	3241.8 ± 858.8	2.9 ± 0.8	1.1 ± 0.3	0.16 ± 0.03	0.11 ± 0.05	0.65 ± 0.18
**Fed**	45	4633.5 ± 2022.7	2646.2 ± 603.1	7.0 ± 3.0	1.3 ± 0.6	0.26 ± 0.10	0.16 ± 0.06	0.63 ± 0.21
Men	23	4607.8 ± 2265.1	2590.7 ± 601.2	5.6 ± 1.1	1.3 ± 0.5	0.25 ± 0.08	0.15 ± 0.07	0.61 ± 0.23
Women	22	4660.4 ± 1787.8	2704.2 ± 613.6	8.4 ± 3.7*	1.2 ± 0.7	0.28 ± 0.12	0.17 ± 0.05	0.65 ± 0.19
**Fed vs. Fast** ratio; 90% CI; *p* value		65.6; 55.7–77.2 *p* = 0.0001	93.7; 85.5–102.7 *p* = 0.2412	227.2; 203.2–254.1 *p* = 0.0001	95.3; 82.8–109.8 *p* = 0.5749	147.1; 134.3–161.0 *p* = 0.0001	151.0; 128.7–177.2 *p* = 0.0001	104.9; 91.1–120.7 *p* = 0.5749
**Rabeprazole (*n*** **= 69)**								
**Fast**	35	971.9 ± 361.2	556.4 ± 176.2	3.7 ± 0.9	1.3 ± 0.5	0.60 ± 0.17	0.35 ± 0.11	0.60 ± 0.20
Men	18	836.3 ± 313.0	479.1 ± 139.7	3.6 ± 0.9	1.2 ± 0.5	0.55 ± 0.11	0.35 ± 0.13	0.64 ± 0.20
Women	17	1115.5 ± 361.4	638.3 ± 177.2	3.7 ± 0.9	1.4 ± 0.5	0.65 ± 0.20	0.35 ± 0.10	0.56 ± 0.20
**Fed**	34	1047.3 ± 382.3	675.8 ± 218.8	7.2 ± 2.6	2.2 ± 1.3	0.91 ± 0.45	0.34 ± 0.18	0.42 ± 0.22
Men	19	1045.8 ± 361.49	634.6 ± 144.8	6.9 ± 3.0	2.3 ± 1.3	0.91 ± 0.52	0.31 ± 0.18	0.38 ± 0.17
Women	15	1049.2 ± 420.3	727.9 ± 284.0	7.6 ± 2.0	2.0 ± 1.3	0.92 ± 0.37	0.38 ± 0.18	0.48 ± 0.27
**Fed vs. Fast** ratio; 90% CI; *p* value		106.7; 91.3–124.8 *p* = 0.4886	121.3; 106.9–137.6 *p* = 0.0132	191.5; 172.2–212.9 *p* = 0.0001	153.1; 126.9–184.7 *p* = 0.0003	144.3; 125.6–165.9 *p* = 0.0001	93.1; 79.5–109.1 *p* = 0.4531	65.3; 54.1–78.8 *p* = 0.0003
**Omeprazole (*n*** **= 36)**								
**Fast**	35	2190.8 ± 2011.5	930.9 ± 434.6	2.0 ± 0.7	1.1 ± 0.6	0.64 ± 0.34	0.53 ± 0.38	0.76 ± 0.29
Men	17	2375.9 ± 2480.1	858.8 ± 432.6	2.1 ± 0.7	1.3 ± 0.8	0.65 ± 0.38	0.50 ± 0.44	0.70 ± 0.29
Women	18	2016.0 ± 1495.4	999.1 ± 437.7	1.9 ± 0.6	1.0 ± 0.4	0.63 ± 0.31	0.56 ± 0.34	0.82 ± 0.29
**Fed**	36#	1928.3 ± 1878.5	682.6 ± 394.1	4.9 ± 1.1	1.3 ± 0.6	1.02 ± 1.07	0.64 ± 0.55	0.67 ± 0.32
Men	18	2135.8 ± 2282.7	725.0 ± 447.6	4.5 ± 0.9	1.3 ± 0.7	0.75 ± 0.52	0.57 ± 0.49	0.69 ± 0.35
Women	18	1720.9 ± 1402.0	640.2 ± 640.1	5.4 ± 1.0	1.3 ± 0.5	1.30 ± 1.38	0.73 ± 0.61	0.65 ± 0.30
**Fed vs. Fast** ratio; 90% CI; *p* value		86.2; 81.2–91.6 *p* = 0.0002	63.0; 55.0–72.2 *p* = 0.0001	262.5; 232.6–296.2 *p* = 0.0001	117.6; 105.6–130.9 *p* = 0.0156	136.1; 105.6–130.9 *p* = 0.0029	116.1; 108.7–124.1 *p* = 0.0006	85.0; 76.4–94.7 *p* = 0.0156

#35 of the subjects participated in both fast and fed omeprazole clinical trials

**Fig. 2:**
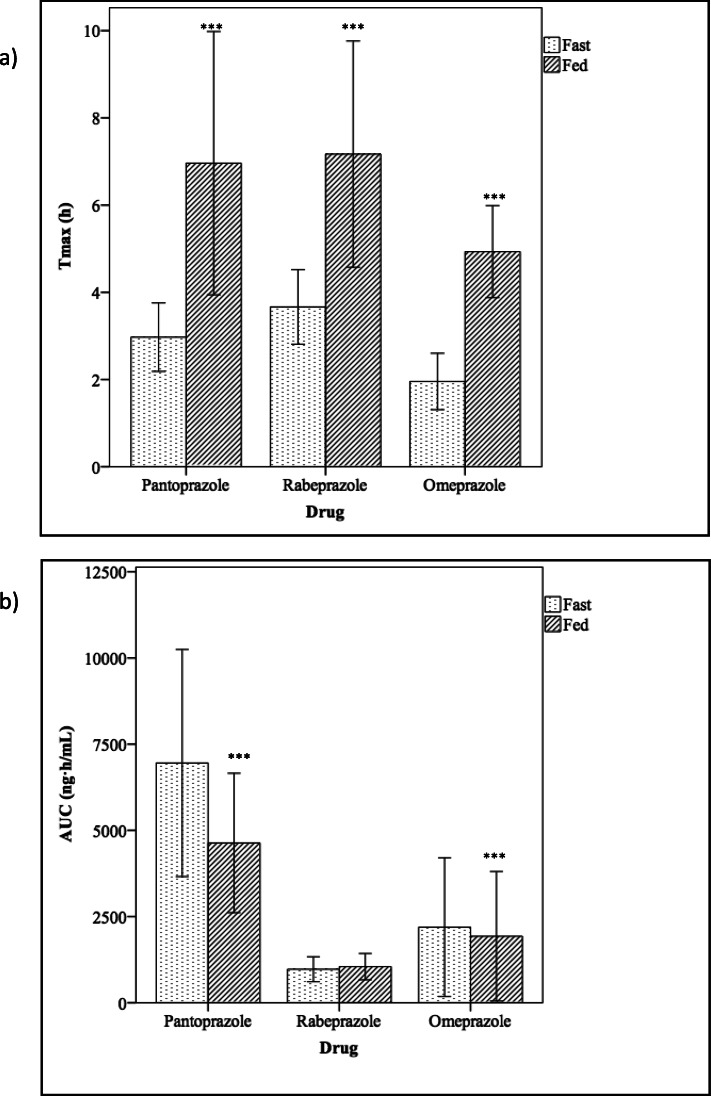
Differences in PPI Tmax (**a**) and AUC (**b**) when administered under fed and fasting conditions. ***p < 0.001. Bars represented as mean and standard deviation

Under fasting conditions, T_max_ was significantly reached earlier for omeprazole (2 h) than for pantoprazole (3 h) and rabeprazole (3.7 h) (*p* ≤ 0.001) (Fig. 2a). In addition, T_max_ was also significantly reached earlier for pantoprazole than for rabeprazole (*p* ≤ 0.001).

T_max_ was delayed when only pharmacokinetic data after food intake were taken into consideration: T_max_ for omeprazole (4.9 h) was earlier than for pantoprazole (7 h) and rabeprazole (7.2 h) (*p* ≤ 0.001), but no differences were found between pantoprazole and rabeprazole (Fig. 2a).

The administration of omeprazole with food delayed its mean T_max_ by about 3 h (*p* ≤ 0.001) and increased the variability of T_max_, with a range of 1–3.5 h under fasting conditions and a range of 1–17 h under fed conditions. Under fed conditions, omeprazole AUC and C_max_ were 12 and 27% lower, respectively (*p* ≤ 0.001), and half-life was 15% higher (*p* ≤ 0.05 and *p* ≤ 0.001, respectively).

Food delayed the pantoprazole T_max_ by around 4 h (*p* ≤ 0.001), with a range of 1.5–5 h under fasting conditions and 1–21 h under fed conditions. In addition, pantoprazole AUC was 33% lower (*p* ≤ 0.001) under fed conditions (Fig. 2b).

Food also increased the rabeprazole T_max_ by about 3.5 h (*p* ≤ 0.001), with a range of 2–7 h under fasting conditions and 3–20 h under fed conditions. The rabeprazole C_max_ and half-life were 21 and 66% higher under fed conditions (*p* ≤ 0.05 and *p* ≤ 0.001, respectively).

When only subjects with CYP2C19*1/*1 were taken into account (*n* = 82; 31 omeprazole, 28 pantoprazole, and 23 rabeprazole) similar results were obtained: food increased variability and delayed T_max_ by 2.7 h for pantoprazole (from 3 to 5.7 h, *p* ≤ 0.001), 3 h for omeprazole (from 1.8 to 4.9 h, *p* ≤ 0.001), and 5.2 h for rabeprazole (from 3.9 h to 9.1 h, *p* ≤ 0.001) (Table 2). In addition, under fed conditions, the rabeprazole half-life was higher (*p* ≤ 0.05); and the omeprazole AUC and C_max_ were lower (*p* ≤ 0.05 and *p* ≤ 0.01, respectively).

**Table 2:** Pharmacokinetic parameters for pantoprazole, rabeprazole, and omeprazole administered under fasting and fed conditions, considering only CYP2C19*1/*1 subjects. Data are expressed as mean ± standard deviation. ^*^p ≤ 0.05 vs. men

	n	AUC_0-∞_ (ng·h/ml)	C_max_ (ng/ml)	T_max_ (h)	t_**1/2**_ (h)	Vd/F (l/kg)	Cl/F (L/h·kg)	Ke
**Pantoprazole (*n*** **= 28)**								
**Fast**	14	6085.3 ± 2922.6	2539.7 ± 836.3	3.0 ± 0.7	1.2 ± 0.4	0.18 ± 0.03	0.12 ± 0.05	0.64 ± 0.19
Men	8	4780.9 ± 2471.8	2019.8 ± 492.8	3.0 ± 0.7	1.3 ± 0.5	0.20 ± 0.02	0.14 ± 0.06	0.64 ± 0.23
Women	6	7824.6 ± 2702.2	3233.0 ± 685.0	2.9 ± 0.8	1.1 ± 0.2	0.16 ± 0.04	0.11 ± 0.04	0.63 ± 0.12
**Fed**	14	4689.0 ± 2303.3	2660.7 ± 531.2	5.7 ± 1.2	1.2 ± 0.5	0.24 ± 0.05	0.16 ± 0.07	0.67 ± 0.20
Men	8	4520.4 ± 2518.4	2564.3 ± 581.1	5.3 ± 1.1	1.2 ± 0.6	0.23 ± 0.03	0.15 ± 0.07	0.67 ± 0.25
Women	6	4913.8 ± 2192.7	2789.3 ± 475.5	6.2 ± 1.1	1.1 ± 0.3	0.25 ± 0.06	0.17 ± 0.08	0.68 ± 0.14
**Fed vs. Fast** ratio; 90% CI; *p* value		74.8; 55.8–100.1 *p* = 0.1010	103.5; 91.3–117.2 *p* = 0.6431	192.2; 166.5–221.9 *p* = 0.001	95.6; 76.7–119.0 *p* = 0.7262	127.5; 113.2–143.6 *p* = 0.0018	129.1; 96.7–172.3 *p* = 0.1439	104.6; 84.0–130.3 *p* = 0.7262
**Rabeprazole (*n*** **= 23)**								
**Fast**	14	901.2 ± 234.6	536.3 ± 138.2	3.9 ± 0.8	1.3 ± 0.6	0.60 ± 0.11	0.37 ± 0.09	0.63 ± 0.20
Men	7	870.2 ± 295.6	512.2 ± 146.8	3.9 ± 1.0	1.5 ± 0.7	0.59 ± 0.10	0.33 ± 0.10	0.57 ± 0.25
Women	7	932.2 ± 172.0	560.4 ± 135.8	3.8 ± 0.7	1.1 ± 0.2	0.61 ± 0.12	0.41 ± 0.07	0.68 ± 0.13
**Fed**	9	968.9 ± 387.5	606.3 ± 206.8	9.1 ± 3.3	2.0 ± 0.9	0.92 ± 0.34	0.38 ± 0.18	0.45 ± 0.23
Men	4	999.7 ± 322.7	572.2 ± 215.1	10.3 ± 5.1	2.5 ± 0.7	1.02 ± 0.39	0.30 ± 0.11	0.30 ± 0.11
Women	5	944.2 ± 469.6*	633.6 ± 220.7	8.2 ± 0.6	1.5 ± 0.9*	0.85 ± 0.32	0.45 ± 0.20*	0.57 ± 0.25*
**Fed vs. Fast**		104.5; 82.7–131.9 *p* = 0.7488	111.7; 89.5–139.2 *p* = 0.3987	228.8; 187.0–278.9 *p* = 0.0001	152.0; 113.4–203.7 *p* = 0.023	147.6; 121.6–179.3 *p* = 0.0025	94.7; 75.1–119.5 *p* = 0.6913	65.8; 49.1–88.2 *p* = 0.023
**Omeprazole (*n*** **= 36)**								
**Fast**	15	1709.0 ± 1299.6	859.5 ± 394.9	1.8 ± 0.6	1.0 ± 0.4	0.69 ± 0.38	0.57 ± 0.39	0.81 ± 0.23
Men	8	1474.9 ± 1403.1	654.1 ± 305.6	2.0 ± 0.7	0.9 ± 0.4	0.68 ± 0.46	0.62 ± 0.44	0.82 ± 0.25
Women	7	1976.5 ± 1219.8	1094.3 ± 366.6	1.5 ± 0.3	1.0 ± 0.3	0.69 ± 0.46	0.53 ± 0.36	0.79 ± 0.23
**Fed**	16#	1523.3 ± 1290.5	604.4 ± 362.6	4.7 ± 1.0	1.0 ± 0.4	0.77 ± 0.43	0.62 ± 0.41	0.80 ± 0.36
Men	10	1409.7 ± 1162.1	582.8 ± 419.1	4.3 ± 1.0	1.0 ± 0.4	0.73 ± 0.38	0.63 ± 0.49	0.84 ± 0.39
Women	6	1712.7 ± 1250.2	640.4 ± 275.1	5.4 ± 0.4	1.1 ± 0.4	0.84 ± 0.54	0.57 ± 0.29	0.72 ± 0.32
**Fed vs. Fast** ratio; 90% CI; *p* value		88.5; 82.4–95.1 *p* = 0.0101	64.3; 52.5–78.7 *p* = 0.0019	278.3; 227.7–340.1 *p* = 0.0001	104.8; 87.7–125.3 *p* = 0.6457	118.9; 93.3–151.5 *p* = 0.2271	115.3; 105.3–126.2 *p* = 0.0159	95.4; 79.8–114.0 *p* = 0.6457

#15 of the subjects participated in both fast and fed omeprazole clinical trials

### Role of sex in the pharmacokinetics of PPIs

There were no differences in the pharmacokinetic parameters between men and women for the three drugs, but sex affected the pantoprazole T_max_ under fed conditions that was higher in women (*p* ≤ 0.05) (Table 1).

When only CYP2C19*1/*1 subjects are taken into account, under fed conditions rabeprazole AUC was higher (*p* ≤ 0.05) and half-life was lower in women (*p* ≤ 0.05).

## Discussion

Our data showed that food delays the absorption of all PPIs by a mean of 3–4 h. This finding agrees with those of previous studies for esomeprazole [13], tenatoprazole [14], and rabeprazole [15].

The effectiveness of the initial antisecretory action of a PPI depends on factors such as timing in relation to meals [16]. Because of their short half-lives, PPIs are best administered before a meal or ideally after a period of fasting [17] to ensure that the proton pumps are maximally activated when the drug is available in plasma [16]. Nevertheless, as shown in a study on physician prescription patterns, there is much confusion about the optimal time to take a PPI in relation to meals [17].

AUC is the primary pharmacokinetic parameter to determine the antisecretory effect on omeprazole that is independent on C_max_ [18]. Meals with a high fat content slow gastric emptying [19], thus delaying absorption of the drug and lowering AUC and C_max_ values [20]. In our opinion, a low-fat breakfast would also influence PPI absorption, however, the effect might be less noticeable and maybe not clinically relevant. In this regard, the presence of food has been reported to reduce the C_max_ and AUC of omeprazole [21] and esomeprazole [13]. Thomson et al. found that the evening meal reduced the tenatoprazole AUC compared with evening administration of the drug under fasting conditions [14]. Our data are in accordance with this finding, since the pantoprazole AUC and omeprazole AUC and C_max_ were significantly lower under fed conditions. A decrease in AUC higher than 20% may be clinically relevant and can influence the effect on acid secretion. However, it should be further evaluated in chronic PPI treatment, since a single-dose study is not the best approach to established the actual correlation.

However, food increased rabeprazole C_max_ and half-life. The metabolism of omeprazole, pantoprazole, and rabeprazole is mediated mainly by CYP2C19 and CYP3A4 [22], but the involvement of CYP3A4 is higher for rabeprazole. The inhibition of CYP3A4 by food may be associate to a lower first-pass metabolism that may explain the increase in C_max_ and half-life when rabeprazole is administered under fed conditions. Although the meals given to the volunteers did not contain grapefruit or other known CYP3A4 inhibitors, other components of food could also inhibit in some extent CYP3A4. Moreover, the difference in C_max_ can also be due to different genetic profile in subjects participating in the fed and fast studies, since it disappears when considering only CYP2C19*1/*1 subjects (see Table 2).

Clearance and volume of distribution cannot be properly calculated when the drug is administered by oral route and we can only calculate these parameters adjusted for bioavailability. In this way, Cl/F is calculated as dose/AUC, and Vd/F as Cl/Ke. So, the differences found in these parameters may reflect the differences in bioavailability when the drug is administered with food.

Food increased variability in T_max_ for all 3 drugs, delaying absorption around 3 to 4 h and until 20 h in some subjects. This can be related with the different effect of a high-fat meal on stomach empting and CYP3A4 inhibition in each subject.

Food intake increases the gastric pH that activates ATPase molecules, thus resulting in acid secretion [2]. PPIs require secretion of acid for activation and binding to ATPase molecules [13]; therefore, food may affect the pharmacokinetics and pharmacodynamics of PPIs [3, 4].

Because of the direct relationship between plasma AUC and the antisecretory effects of PPIs [23, 24], it might be expected that administration of PPIs with food would decrease acid suppression. In this respect, Andersson et al. reported that inhibition of intragastric acid secretion by esomeprazole increases with higher exposure (AUC) [23]. Therefore, a higher AUC correlates with higher efficacy. For this reason, in the case of pantoprazole and omeprazole, since food decreases the AUC, it would be better to administer these drugs under fasting conditions. However, Iwata et al. observed that pre-dinner administration of PPIs could increase their efficacy in patients with gastroesophageal reflux disease [25].

Since findings are contradictory, some authors state that food did not affect PPIs. In their review, Swan et al. reported that the bioavailability of rabeprazole was not influenced by co-ingestion of food [26]. Junghard et al. found that food decreased AUC and C_max_ but had no effect on the percentage of time that intragastric pH was > 4.0, because of the more extended plasma concentration profile (longer duration with esomeprazole) [24]. Huber et al. observed that concomitant intake of a standard breakfast with pantoprazole (40 mg) had no effect on bioavailability [27]. In this regard, our study sheds light to this controversy, since we found a clear influence of food intake in omeprazole, rabeprazole and pantoprazole pharmacokinetic parameters.

In our opinion, based on our results, omeprazole and pantoprazole drug label should include the following sentence: “As food delays the absorption of PPIs around 3 to 4 hours and decreases their bioavailability, it would be better to administer these drugs under fasting conditions.”

Finally, the effect of sex was analysed because all factors that may influence pharmacokinetics must be taken into account. However, the differences found in AUC for rabeprazole are very small (around 5%), so it is not expected to be related to a different clinical effect.

## Conclusion

In conclusion, administration of PPIs with food delays absorption around 3 to 4 h and increases their variability. Food also decreases oral exposure of omeprazole and pantoprazole. Consequently, it would be better to administer PPIs under fasting conditions to improve their efficacy.

## Data Availability

The dataset supporting the conclusions of this article is available under the petition to the corresponding author.

## Abbreviations

AUC: Area under the concentration-time curve
BMI: Body Mass Index
Cmax: Maximum concentration
CYP: Cytochrome P450
ECG: Electrocardiogram
EMA: European Medicines Agency
FDA: US Food and Drug Administration
PPI: Proton pump inhibitor
Tmax: Time to reach the maximum concentration
